# OVA-Experienced CD4^+^ T Cell Transfer and Chicken Protein Challenge Affect the Immune Response to OVA in a Murine Model

**DOI:** 10.3390/ijms22126573

**Published:** 2021-06-18

**Authors:** Ewa Fuc, Dagmara Złotkowska, Ewa Wasilewska, Barbara Wróblewska

**Affiliations:** Department of Food Immunology and Microbiology, Institute of Animal Reproduction and Food Research, Polish Academy of Sciences, J. Tuwima 10 Str., 10-748 Olsztyn, Poland; e.fuc@pan.olsztyn.pl (E.F.); b.wroblewska@pan.olsztyn.pl (B.W.)

**Keywords:** adoptive transfer, allergy, ovalbumin, chicken meat

## Abstract

Chicken meat is often a major component of a modern diet. Allergy to chicken meat is relatively rare and occurs independently or in subjects allergic to ovalbumin (OVA). We examined the effect of adoptive transfer of OVA-CD4^+^ T cells on the immune response to OVA in mice fed chicken meat. Donor mice were injected intraperitoneally with 100 µg of OVA with Freund’s adjuvant two times over a week, and CD4^+^ T cells were isolated from them and transferred to naïve mice (CD4^+^/OVA/ChM group), which were then provoked with OVA with FA and fed freeze-dried chicken meat for 14 days. The mice injected with OVA and fed chicken meat (OVA/ChM group), and sensitized (OVA group) and healthy (PBS group) mice served as controls. Humoral and cellular response to OVA was monitored over the study. The CD4^+^/OVA/ChM group had lowered levels of anti-OVA IgG and IgA, and total IgE. There were significant differences in CD4^+^, CD4^+^CD25^+^, and CD4^+^CD25^+^Foxp3^+^ T cells between groups. OVA stimulation decreased the splenocyte proliferation index and IFN-γ secretion in the CD4^+^/OVA/ChM group compared to the OVA group. IL-4 was increased in the OVA/ChM mice, which confirms allergenic potential of the egg–meat protein combination. Transfer of OVA-experienced CD4^+^ T cells ameliorated the negative immune response to OVA.

## 1. Introduction

After milk, eggs are the leading source of allergens in children’s diets and are one of the most common reasons for severe anaphylaxis [1]. The main allergens defined are ovalbumin (OVA) and ovomucoid (egg white Gal d 2 and Gal d 1, respectively). Cross-reactivity was found between these proteins from different avian species, like turkey or duck [2]. In egg yolk, other proteins able to bind IgE, i.e., α-livetin, also known as chicken serum albumin (Gal d 5), and yolk glycoprotein 42 (YGP42; Gal d 6) were determined [3]. Gal d 5 is implicated in bird–egg syndrome and is found in chicken meat [1,3].

Allergy is a significant public health problem affecting 3–10% of adults and 8% of children worldwide. Allergy to chicken meat affects only 0.6 to 5% of food-allergic subjects, therefore, it is rare compared to more common food allergies, such as to milk, eggs, or fish [4]. Chicken meat allergy may be either primary or, when related to bird–egg syndrome, secondary [5]. Bird–egg syndrome is a respiratory allergy to bird feathers, dander, and meat that develops into a concomitant egg yolk allergy [6]. Some cases of primary chicken allergy reported in the literature present severe clinical symptoms following chicken meat consumption without prior allergy to eggs [7,8]. Clinical symptoms of chicken meat allergy are generally relatively mild and can include contact reactions, oral allergy syndrome, or regular systemic reactions (skin and gastrointestinal reactions) [5,6]. Clinical and demographic studies showed that patients diagnosed with bird–egg syndrome have a strong IgE response to egg white’s main allergen (OVA; Gal d2) and chicken meat [9]. Patients with genuine chicken meat allergy also showed the presence of IgE binding to egg white [5]. To avoid allergic reactions, the elimination of chicken meat from the diet is the suggested treatment. However, poultry meat is a valuable dietary component because of its highly digestible proteins, B-group vitamins (mainly thiamin, vitamin B6, and pantothenic acid), and minerals (such as iron, zinc, and copper). Thus, eliminating chicken meat from the diet may result in a shortage of valuable nutrients [10]. Moreover, poultry meat is often the dietary base of specific consumer groups such as children and the elderly, and during certain conditions like pregnancy and breastfeeding [11]. Furthermore, many experts recommend that chicken is the first meat introduced into children’s diets following the breastfeeding stage [11]. Therefore, more research into the immune mechanism underlying the reaction to this potential food allergen is required.

The induction of peripheral tolerance to food allergens may constitute a disease-modifying treatment for allergic patients. Smaldini et al. [12] confirmed that regulatory T cells (Tregs) play an essential role in resolving food allergies. They found that the adoptive transfer of CD4^+^CD25^+^Foxp3^+^ Treg cells induced a protective mechanism and that the depletion of CD25^+^ T cells resulted in pronounced disease exacerbation. In turn, Haczku et al. [13] demonstrated that the adoptive transfer of OVA-specific CD4^+^ T cells into recipient mice induced modulation of the immune response and airway inflammation because of airway wall infiltration by eosinophils in the CD4 transfer mice [13]. In another study, OVA-specific Treg cells were transferred to naïve mice, resulting in suppression of the anaphylactic response to OVA [14]. Wang et al. [15] demonstrated that an adoptive transfer of CD4^+^CD25^+^ Tregs reduced the inflammatory response. Therefore, the transfer of specific Tregs may be a beneficial tool in the inhibition of immune response to OVA.

This study aimed to determine whether chicken meat delivery to OVA-sensitized mice could affect their immune response to OVA. In addition, we hypothesized that OVA-experienced CD4^+^ T cell transfer may exert a modulatory effect on the development of immune response in OVA-sensitized mice fed chicken meat.

## 2. Results

### 2.1. Adoptively Transferred OVA-Sensitized CD4^+^ T Cells Affect the Mouse Humoral Response

**Figure 1:**
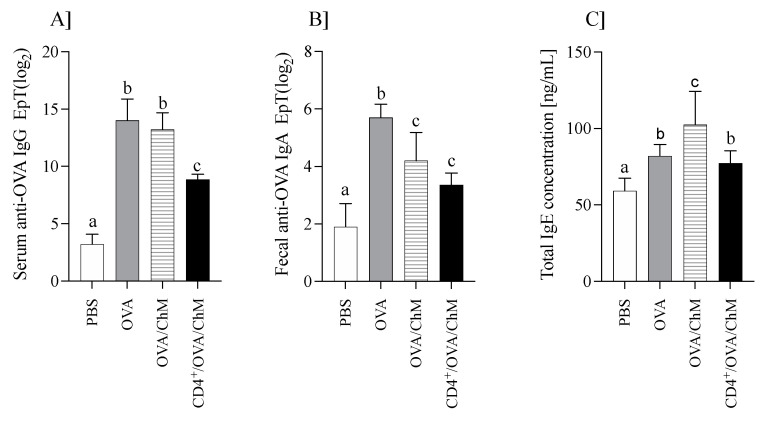
The humoral response in mice of PBS, OVA, OVA/ChM, and CD4^+/^OVA/ChM groups: (**A**) serum anti-OVA IgG; (**B**) fecal anti-OVA IgA; (**C**) total serum IgE concentration. One-way ANOVA with a post hoc Tukey test and a Kruskal–Wallis test was used to determine statistical differences. Bars represent the mean of the group (*n* = 8) ± SD. ^a,b,c^ Mean values with different superscripts are different at *p* ≤ 0.05.

The mouse humoral response (OVA-specific IgG and IgA, and total IgE) is shown in Figure 1.

Immunization of mice with OVA increased anti-OVA IgG levels (Figure 1A). OVA/ChM and OVA groups showed about four times higher anti-OVA IgG titers than the PBS group, while those of the CD4^+^/OVA/ChM group were only 2.8 times higher (*p* < 0.05 vs. PBS group). Additionally, specific to OVA, IgA titers in fecal extracts were varied in the groups (Figure 1B). The highest level was in the OVA group (2^5.7±0.9^), followed by OVA/ChM (2^4.2±0.98^) and CD4^+^/OVA/ChM (2^3.35±0.42^), compared to 2^1.9±0.91^ in the PBS group (*p* < 0.001).

Total serum IgE levels were determined in all groups (Figure 1C). IgE concentration in the sensitized groups was higher than in the PBS control group (*p* < 0.05). Mice immunized with OVA and fed chicken meat (OVA/ChM group) had the highest total IgE levels of 102 ± 22 ng/mL compared to 77.2 ± 8.1 ng/mL in the CD4^+^/OVA/ChM group (*p* < 0.05). The total IgE concentration in the PBS control group was 59.1 ± 8.4 ng/mL.

The results showed that feeding chicken meat to mice did not increase OVA-specific antibody titers more than in control mice sensitized to OVA. This may indicate the lack of cross-reactions between OVA and meat proteins. In turn, a significant increase in total IgE in the OVA/ChM group compared to the OVA group suggests that proteins from chicken meat induced an immune response [16].

### 2.2. Changes in CD3^+^CD4^+^, CD3^+^CD4^+^CD25^+^, and Foxp3^+^ Populations Induced by the Tested Proteins

**Figure 2:**
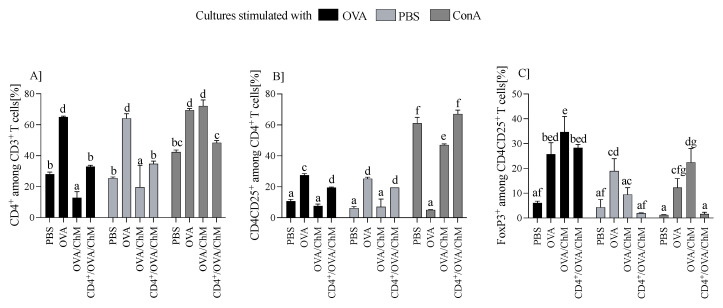
Distribution of T cell subpopulations among lymphocytes from PBS, OVA, OVA/ChM, and CD4^+^/OVA/ChM groups after ex vivo culture in the presence of ovalbumin (OVA; black bars), phosphate-buffered saline (PBS; light gray bars), or concanavalin A (ConA; dark gray bars; a positive assay control). The gating tree was as follows: lymphocytes were gated from FSC/SSC; next, CD4^+^ cells were gated among CD3^+^ T cells (**A**), and then CD3^+^CD4^+^CD25^+^ among the CD4^+^ cells (**B**); next, the Foxp3^+^ population was gated among CD3^+^CD4^+^CD25^+^ cells (**C**). Each sample was assessed in triplicate and 50,000 events were collected for each assay. Two-way ANOVA with a post hoc Tukey test was used to determine statistical differences. Bars represent the mean of each group (*n* = 3) ± SD. ^a,b,c,d,e,f,g^ Mean values with different superscripts are different at *p* ≤ 0.05.

Splenocytes were cultured ex vivo with OVA, ConA, or PBS (controls), and changes in cellular response were measured by monitoring the percentage of CD3^+^, CD4^+^, CD4^+^CD25^+^, and Treg cells. We found that OVA has a significant effect on T cell populations (Figure 2). Splenocytes from the OVA group induced the highest percentage of CD4^+^ T cells (64.7 ± 0.87) compared to other groups (*p* < 0.001). Compared to the OVA group, cultures from the CD4^+^/OVA/ChM group had about 50% fewer CD3^+^CD4^+^ cells (32.9 ± 0.87), and from the OVA/ChM group, about 80% (12.7 ± 3.98), which was the lowest determined level of these cells (*p* < 0.001 vs. other groups; Figure 2A). A similar trend was observed in populations of CD3^+^CD4^+^CD25^+^ T cells, of which the OVA group showed the highest percentage of these cells (27.47 ± 1.06), with CD4^+^/OVA/ChM being lower (19.5 ± 0.35), and OVA/ChM the lowest (7.6 ± 1.2) (*p* < 0.001; Figure 2B). The percentages of Foxp3^+^ Treg lymphocytes were 28.3 ± 1.3 in the CD4^+^/OVA/ChM group and 34.7 ± 6.2 in the OVA/ChM group, both significantly higher than in the PBS group (6.05 ± 0.76) (*p* < 0.001; Figure 2C).

### 2.3. Adoptive Transfer of the CD4^+^ T Cells Altered Lymphocyte Proliferation Capacity

**Figure 3:**
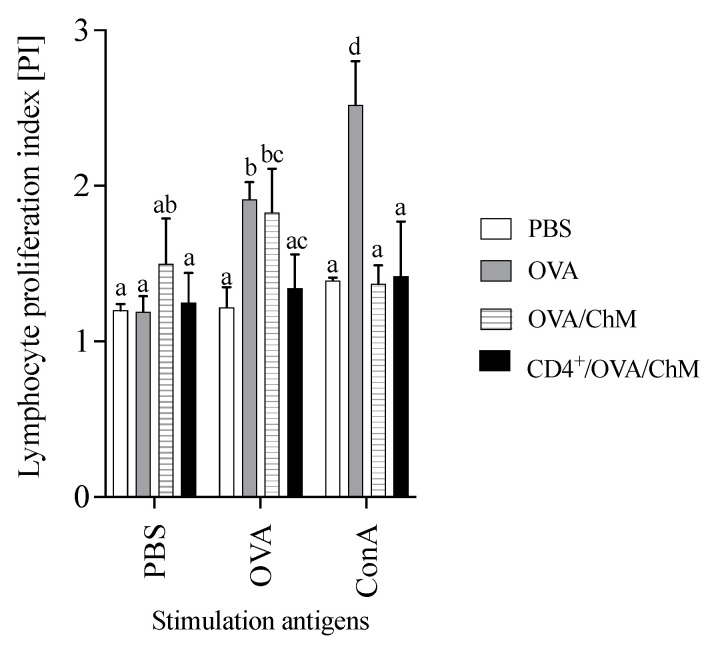
Proliferation index (PI) of lymphocytes from PBS, OVA, OVA/ChM, and CD4^+^/OVA/ChM groups: unstimulated (PBS), and stimulated with ovalbumin (OVA) or concanavalin A (ConA; assay positive control). Two-way ANOVA with a post hoc Tukey test was used to determine statistical differences. The bars represent the mean of the group (*n* = 3) ± SD. ^a,b,c,d^ Mean values with different superscripts are different at *p* ≤ 0.05.

To verify the effect of adoptively transferred CD4^+^ T cells on the cellular response to OVA in experimental groups, a proliferation index (PI) was carried out in the culture with stimulation (Figure 3). After OVA stimulation, the PI of splenocytes from the CD4^+^/OVA/ChM group was lower (1.34) than that of the OVA group (1.91) (*p* < 0.05). The unstimulated cells (PBS added to the culture) in the OVA/ChM group had a PI of 1.50, while the PIs of cells from the PBS and CD4^+^/OVA/ChM groups were lower (1.20 and 1.25, respectively), but without any statistical differences.

### 2.4. Changes in Cytokine Secretion Following CD4^+^ T Cell Transfer

**Figure 4:**
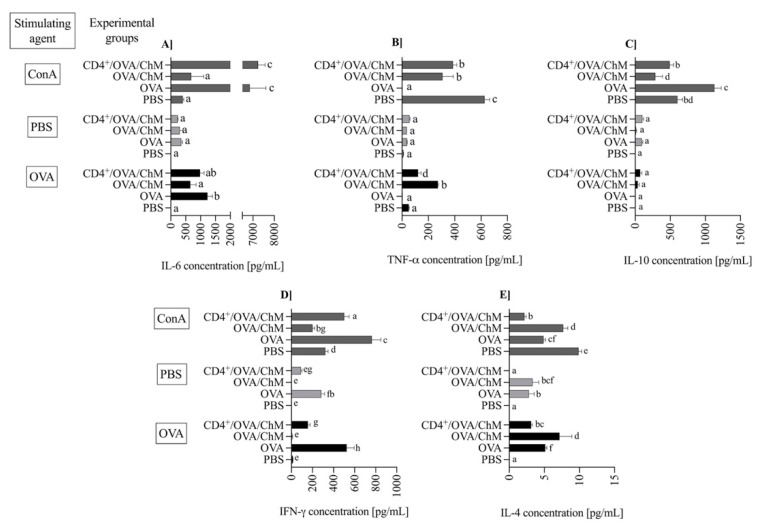
The concentration of cytokines: IL-6 (**A**), TNF-α (**B**), IL-10 (**C**), IFN-γ (**D**) and IL-4 (**E**) in splenocyte cultures from PBS, OVA, OVA/ChM, and CD4^+^/OVA/ChM groups: unstimulated (PBS; light gray bars), and stimulated with ovalbumin (OVA; black bars) or concanavalin A (ConA; dark gray bars; assay positive control). Two-way ANOVA with a post hoc Tuckey test was used to determine differences between groups and treatments. The bars represent the mean of the group (*n* = 3) ± SD. ^a,b,c,d,e,f,g^ Mean values with different superscripts are different at *p* ≤ 0.05.

The splenic lymphocytes isolated from the experimental groups were cultured either unstimulated (PBS) or stimulated with OVA or ConA (an assay positive control). The levels of secreted cytokines were evaluated after 120 h of incubation (Figure 4).

Pro- and anti-inflammatory cytokines are mediators used to track food allergy development. In the experiment, the concentration of IL-6 during OVA stimulation was significantly higher in the OVA (1235 ± 162 pg/mL) and CD4^+^/OVA/ChM (981 ± 136 ng/mL) groups in comparison to PBS or OVA/ChM groups (9.5 ± 1.4 and 657 ± 191, respectively; *p* < 0.01; Figure 4A). At the same time, low levels of TNF-α were observed in OVA and CD4^+^/OVA/ChM groups (*p* < 0.01 vs. OVA/ChM group; Figure 4B). The concentration of multifunctional cytokine IL-10 was slightly elevated in CD4^+^/OVA/ChM lymphocyte culture (72 ± 33 pg/mL) compared to the OVA/ChM group (38 ± 20 pg/mL; Figure 4C).

IFN-γ exerts an inhibitory effect on cytokines released by Th2 cells and thus modulates inflammatory response. We observed the highest concentration of IFN-γ after OVA stimulation in the OVA control group (525 ± 71 pg/mL; Figure 4D), accompanied by secretion of 5.13 ± 0.23 pg/mL of IL-4 (Figure 4E). In the OVA/ChM group, IFN-γ concentration was low, 7.0 ± 4.1 ng/mL, and corresponded to a high concentration of IL-4, 7.2 ± 1.7 ng/mL. Lymphocytes from the CD4^+^/OVA/ChM group released significant amounts of interferon (156 ± 21 pg/mL; *p* < 0.01 vs. other groups), and only 4.3 ± 0.19 pg/mL of IL-4 (*p* < 0.01).

## 3. Discussion

Food hypersensitivity results from an imbalance between Th2 and Th1 responses [17]. Recently, researchers have investigated the possibility of redirecting the Th2 response in favor of the Th1 response, which could reduce the occurrence of atopy [18]. Modulation of the immune response via the adoptive transfer of sensitized lymphocytes has been described previously [13,15,19,20]. However, the studies were mainly focused on the observation of the respiratory system. One study showed that the adoptive transfer of specific splenocytes could induce a response to an aerosol version of OVA in naïve mice [21]. Other studies have focused on the adoptive transfer of Tregs as an opportunity to suppress allergic reactions leading to airway inflammation. This is because airway hypersensitiveness and total IgE levels relate to an increase in Treg cells, which appear to reduce these allergy symptoms [22].

This study presented evidence through a novel approach that adoptive transfer of OVA-experienced CD4^+^ T cells can modulate the immune response to OVA in mice intraperitoneally treated with OVA and fed with chicken meat. We observed significant differences between the groups in both humoral and cellular responses. The CD4^+^/OVA/ChM group exhibited a lower humoral response (IgE, IgA, and IgG) than the OVA and OVA/ChM groups. The levels of anti-OVA IgG and secretory anti-OVA IgA were lower in the CD4^+^/OVA/ChM group than in the OVA/ChM group, though they were about two times higher than in the PBS group. This suggests that CD4^+^ T cell transfer enhanced the mechanisms of a protective process. IgE level in the CD4^+^/OVA/ChM group was reduced by 25% compared to the OVA/ChM group, though there was no difference when compared to the OVA group. Nevertheless, IL-4 concentration, a key cytokine in the pathomechanism of food allergy, was significantly lower in the former group, confirming the protective effect of the transferred CD4^+^ T cells. IgE synthesis requires signals from IL-4 and CD40 ligand expressed on activated CD4^+^ T cells. Both IL-4 and CD4^+^ T cells were significantly lowered in the CD4^+^/OVA/ChM group. Similarly, we found lower concentrations of IFN-γ in this group although, generally, in allergy the concentration of this marker is characterized by a large scatter of results. In line with this, the significantly higher level of total IgE in the sera of the OVA/ChM group compared to the control OVA group confirms the antigenic properties of chicken meat proteins, i.e., serum albumin (Gal d5) [1]. The observed reduction in IgE is particularly important, as this immunoglobulin is considered the primary indicator of food allergy [23,24]. Allergy studies using animal models have shown that allergic reactions in mice are characterized by an increase in total IgE level compared to control animals [25]. Our results showed that adoptive transfer of OVA-sensitized lymphocytes reduced the humoral response to OVA in the CD4^+^/OVA/ChM group compared to the OVA/ChM group. Therefore, it could be a promising new method to increase tolerance to allergens in subjects suffering from egg and chicken meat allergy.

The CD4^+^ T cell-mediated cellular response plays a crucial role in an allergic reaction, which involves lymphocyte activation and cytokine production [26]. Splenocytes obtained from OVA and CD4^+^/OVA/ChM groups showed a changed contribution of lymphocyte subpopulations. OVA stimulation activated a higher percentage of CD4^+^CD25^+^ Treg cells in the CD4^+^/OVA/ChM group compared the OVA/ChM group (Figure 2C and Figure 3C). Induced Tregs are extremely important in mitigating immune response to food allergens [23,27]. In addition, stimulation of cells with OVA resulted in decreased proliferative capacity of the lymphocytes in the CD4 transfer mice compared to the OVA mice. This indicates that adoptively transferred OVA-sensitized CD4^+^ T cells inhibited the immune response to OVA in the mice tested and confirms that transferred sensitized donor CD4^+^ T cells have the potential to modulate the host immune response. Evidence suggests that the proliferative response of PMBC lymphocytes to OVA or BSA in children with egg or milk sensitivities is significantly higher than that of healthy children [27]. Hoffman et al. [28] presented an observational study that compared patients with IgE-mediated allergy to a control group, showing increased proliferative response to milk antigens in the first group. The secretion of Th1 or Th2 cytokines defines whether the response to a potential allergen is tolerance or an allergic reaction [29,30,31]. In the current study, splenocytes isolated from the tested groups secreted varied amounts of cytokines. Following stimulation with OVA, the CD4^+^/OVA/ChM group splenocytes secreted larger amounts of IFN-γ than those of the OVA/ChM group (*p* < 0.05). As described above, CD4^+^ T cell transfer decreased the production of specific antibodies (Figure 1), thus inducing anti-inflammatory processes, as indicated by a decrease in IL-4 secretion in the CD4^+^/OVA/ChM group by 55% compared to the OVA/ChM group, and by 27% compared to OVA group. In turn, the higher concentrations observed in the OVA/ChM group are due to the induction of reactive cells by the combination of OVA and chicken meat proteins. As IL-4 plays a role in stimulating the production of both B lymphocytes and IgE, a decrease in its level indicates a shift in the host immune response toward tolerance.

IFN-γ was found in cultures of splenocytes obtained from the CD4^+^/OVA/ChM and OVA groups. The presence of IFN-γ increases the expression of MHC II molecules and stimulates the cross-reactive presentation of antigens to T cells [32]. Further, IFN-γ is involved in the differentiation of B lymphocytes and the release of phagocytic antibodies [33]. The presence of IFN-γ may result from the adoptive transfer of the OVA-sensitized CD4^+^ T lymphocytes, which proves that transfer the CD4^+^ cells transferred their properties to naïve mice and modulated the allergic response by inhibiting the Th2 response and influencing IL-4 release [34].

The multifunctional cytokine IL-10 is primarily produced by stimulated T cells and suppresses strong inflammatory reactions [35,36]. Marconi et al. [37], in their research with human serum, demonstrated that, together with IL-4, it induced IgA production, which is in line with our studies. The development of immune response involves many pro- and anti–inflammatory mediators. IL-6 is the T cell- and macrophage-derived cytokine presenting both properties. It induces inflammation by releasing acute phase proteins and neutrophils in the bone marrow. It also suppresses inflammation by inhibiting TNF-α and inducing IL-10 [38]. Dienz et al. [39] reported that the presence of IL-6 is required for IgG_1_ production; on the other hand, the reduced IL-6 levels are responsible for ineffective class-switching from IgE to IgG, leading to increased allergic sensitization. In our study, we observed an increase in IL-6 concentration in OVA-stimulated splenocytes in all experimental groups compared to PBS (*p* < 0.05). At that time, however, the highest concentration of TNF-α was determined in the OVA/ChM group (*p* < 0.05). This fact is consistent with the observed higher humoral response in the OVA/ChM group and the high proliferative capacity of splenocytes from these mice. This confirms the possible immunogenic potential of chicken meat for subjects sensitive to OVA.

To sum up, to the best of our knowledge, this is the first report of a study combining adoptive transfer of OVA-immunized CD4^+^ lymphocytes, OVA sensitization, and chicken meat feeding to show the ability of the modulation of the immune response to OVA. Transfer of the CD4^+^ T cells lowered the humoral response of mice, silencing Th2-related mediators (specific to OVA IgG and IgA and total IgE, *p* < 0.001), increasing the percentage of CD4^+^ lymphocytes, and changing the concentration of secreted cytokines compared to both or one of the OVA-treated control groups. The results demonstrated that chicken meat proteins enhanced the immune response to OVA. Adoptive transfer of sensitized lymphocytes impacted the immune response and reduced the level of allergy markers. This indicates that CD4^+^ T cells play a pivotal role in the induction of immune tolerance to egg allergens. However, further studies are needed to understand the exact mechanism of action of lymphocyte transfer in the treatment of food hypersensitivity, including the identification of T-helper cell subpopulations showing the observed protective effect. It will also be valuable to extend the study to identify chicken meat proteins and peptides that induce T cell response and to evaluate their cross-reactivity with egg proteins.

## 4. Materials and Methods

### 4.1. Animals

Female BALB/cCmdb mice were purchased from the Center of Experimental Medicine in Białystok, Poland. All the mice were housed in a pathogen-free barrier facility. Animals were maintained according to the animal care guidelines of the Local Ethical Committee for Animal Experiments in Olsztyn (permission #43/2015/N).

### 4.2. Protein and Chicken Meat Used for Mouse Treatment

Ovalbumin was purchased from Sigma-Aldrich (Sigma-Aldrich, St. Louis, MO, USA). The chicken meat was bought at the local market. The meat was cooked for 15 min, cooled, cut into small pieces, and freeze-dried. The protein concentration was determined by the Kjeldahl method [40,41]. Samples of chicken meat (3 mg protein/mouse) were dissolved in 200 μL of PBS and used for the animal treatments.

### 4.3. Experimental Design

**Figure 5:**
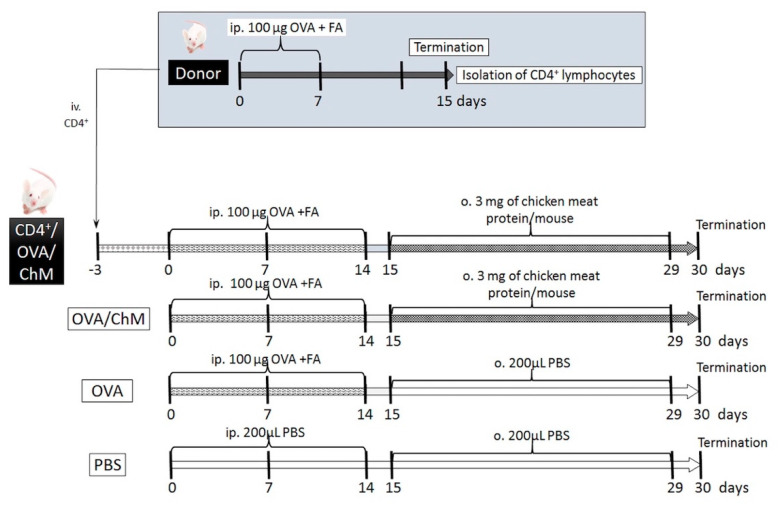
Experiment scheme. Abbreviations used: OVA—chicken egg ovalbumin; FA—Freund’s adjuvant; ChM—chicken meat; ip.—intraperitoneally; o.—oral; iv.—intravenously; PBS—phosphate buffered saline.

The experiment involved in vivo sensitization of CD4^+^ T cells to OVA in donor mice, followed by their transfer to naïve mice, and further mouse provocation with OVA and chicken meat. We hypothesized that the adoptively transferred OVA-experienced CD4^+^ T cells in naïve mice would decrease the development of immune response to OVA in mice sensitized to OVA and treated chicken meat. In this context, the following experimental groups were formed (Figure 5):

- CD4^+^/OVA/ChM group—received intravenous (iv.) transfer of OVA-experienced CD4^+^ T cells, then the mice were injected intraperitoneally (ip.) with OVA and gavaged with freeze-dried chicken meat solution;
- OVA/ChM group—mice were injected ip. with OVA and gavaged with freeze-dried chicken meat;
- OVA group—injected ip. with OVA;
- PBS group—injected ip. and gavaged with PBS.

#### 4.3.1. Generating OVA-Experienced CD4^+^ T Cells for Transfer

The BALB/c group of mice (*n* = 20, donor mice) were injected with 100 µg OVA dissolved in PBS with Freund’s adjuvant (200 μL total volume) via intraperitoneal injection on days 0 and 7. On day 15, the animals were euthanized with carbon dioxide inhalation, and the spleens and head and neck lymph nodes were harvested to isolate the OVA-specific CD4^+^ T cells. Tissues were homogenized in RPMI 1640 (Sigma-Aldrich, St. Louis, MO, USA) supplemented with 10 nM HEPES (Sigma-Aldrich, St. Louis, MO, USA) and 10 units/mL penicillin–streptomycin solution (Sigma-Aldrich, St. Louis, MO, USA) (incomplete medium, IM). Cells were filtered through an 80 µm nylon filter, and red blood cells were lysed with Red Cells Lysis Buffer (Sigma-Aldrich, St. Louis, MO, USA). Then, the lymphocytes were washed and suspended in 1 mL of IM. The number of cells was calculated using a hemocytometer (Sigma-Aldrich, St. Louis, MO, USA). CD4^+^ T cells (96% pure) were collected using a Dynal Mouse CD4 Negative Isolation Kit (DYNAL^®^, Oslo, Norway) with magnetic beads according to the manufacturer’s instructions. CD4^+^ T cells (5 × 10^5^ cells) were suspended in 100 µL of sterile phosphate-buffered saline (PBS; Sigma-Aldrich, St. Louis, MO, USA) and transferred into naïve mice by intravenous injection (CD4^+^/OVA/ChM group).

#### 4.3.2. Animal Sensitization with OVA and Challenge with Chicken Meat

Mice were divided into 4 experimental groups: PBS, OVA, OVA/ChM, and CD4^+^/OVA/ChM (n = 8/group) (Figure 1). Three days before starting the sensitization, the CD4^+^/OVA/ChM group was injected intravenously (iv.) with OVA-experienced CD4^+^ T cells (see Section 4.3.1). Next, the OVA, OVA/ChM, and CD4^+^/OVA/ChM groups were given intraperitoneal injections of 100 µg OVA in PBS with complete Freund’s adjuvant (1:1; 200 μL in total) on day 0, and boosts on days 7 and 14 using a similar emulsion but based on incomplete FA. Non-sensitized control mice (PBS group) treated with PBS served as a negative control for monitoring the immunization process. Starting from day 15, for the following 2 weeks, the OVA/ChM and CD4^+^/OVA/ChM groups were fed intragastrically with freeze-dried chicken meat with 3 mg of protein per mouse (dissolved in 200 μL PBS), and the OVA and PBS groups received an adequate amount of PBS. On days 15, 22, and 29 of the experiment, 10 μg of cholera toxin were administrated to mice as a mucosal adjuvant. On day 30, mice were euthanized with carbon dioxide inhalation; blood and feces samples were collected, and spleens were taken for lymphocyte isolation. Fecal pellets were extracted with 0.1% NaN_3_ in PBS for 20 min at a temperature of 4 °C and centrifuged at 16,900× g (Eppendorf 5418, Hamburg, Germany), at a temperature of 10 °C for 10 min. Blood was coagulated and centrifuged using the conditions described above. Samples were stored at a temperature of −20 °C until analysis [42].

### 4.4. Lymphocyte Isolation and Culture

Lymphocytes were isolated from spleen as described in Section 4.3.1, but without separation of CD4^+^ cells. Purified spleen lymphocytes (SPLs) were cultured in 96-well plates (1 × 10^6^/well) with 200 µL of the complete medium (CM: RPMI 1640 containing 10% heat-inactivated fetal bovine serum, 1 mM non-essential amino acid, 1mM sodium pyruvate, 1mM HEPES, and 10 units/mL penicillin–streptomycin) and then incubated for 120 h, at 37 °C and 5% CO_2_, in the presence of either concanavalin A (ConA, positive control) (10 µg/mL) or OVA (100 µg/mL). The supernatants were collected and stored at −80 °C until analysis, and lymphocytes were harvested for phenotyping by flow cytometry [43].

### 4.5. Phenotyping Lymphocytes

Lymphocytes were labeled with APC Cy7 rat anti-mouse CD4 (Cat. No. 552051; BD Biosciences, San Diego, CA, USA), PE rat anti-mouse CD3 (555275; BD Biosciences, San Diego, CA, USA), and FITC rat anti-mouse CD25 (553071; BD Biosciences, San Diego, CA, USA) for 15 min at 4 °C. Then, the cells were washed with FACS buffer (PBS with 5% FBS) and fixed with 2% paraformaldehyde. Next, the lymphocytes were permeabilized with ice-cold methanol at room temperature (RT) for 20 min, washed, and labeled with Alexa Fluor rat anti-mouse Foxp3 (560401; BD Biosciences, San Diego, CA, USA). The cells were examined on a BD LSRFortessa cell analyzer (BD Biosciences, San Jose, CA, USA), and the obtained data were analyzed using BD FACSDIVA software version 7.0 (BD Bioscience, San Jose, CA, USA) [44].

### 4.6. Cytokine Determination

Cytokine levels in the splenic culture supernatants were determined using the BD Cytometric Bead Array Mouse Inflammation Kit (560485, BD Biosciences, San Diego, CA, USA) according to the manufacturer’s protocol. Theoretical limits of detection provided by the manufacturer are IL-2 0.1; IL-4 0.03; IL-6 1.4; IFN-γ 0.5; TNF-α 0.9; IL-17A 0.8; and IL-10 16.8 pg/mL. Results were analyzed with FCAP Array software version 3.0 (BD Biosciences, San Jose, CA, USA) and presented as a mean of the group ± SD.

### 4.7. Lymphocyte Proliferation Index (PI)

Splenocytes suspended in CM (10^7^/mL) were labeled with 1.1 µL of 5mM CFSE solution. Next, the lymphocytes were incubated for 5 min at RT in the dark. Following labeling, the cells were washed two times with PBS with 5% FBS and one time in PBS with 1% FBS and then centrifuged at 300× *g* for 5 min at 20 °C. Then, the cells were plated on 96-multiwell plates at a concentration of 1 × 10^6^ cells/well per 200 µL of CM and stimulated with ConA (1 µg/well) or with OVA (10 µg/well) and incubated at 37 °C with 5% CO_2_. Following 120 h of culture, the cells were collected and additionally stained with PerCP-Cy5.5 anti-mouse CD4 (550954; BD Biosciences, San Diego, CA, USA) and analyzed using the BD LSRFortessa flow cytometer. FlowJo ^TM^ LLC software version 10.7.1 (BD Biosciences, San Jose, CA, USA) was used for the analysis of the results.

### 4.8. Measurement of Total IgE Concentration and Anti-OVA IgA and IgG Titers

Total IgE was determined by sandwich ELISA, using a commercial test (Cat. No. 157718; Abcam, Cambridge, UK) following the manufacturer’s protocol. Specific to OVA, serum IgA and IgG and feces IgA were measured by indirect ELISA (16). The antigen (1 µg/well in 10 mM PBS, pH 7.4) was coated onto 400 Nunlock microplates (Greiner Bio-One Gmbh, Frickenhausen, Germany) and incubated at 37 °C for 1 h. Next, the plates were blocked with 1.5% gelatin diluted in PBS and incubated under the same conditions. Following blocking, the plates were washed with PBST (PBS + 0.05% Tween 20). Serial dilutions of serum and feces extracts (50 µL) were added to the plates and incubated for 1 h at 37 °C. After a washing step, the plates were incubated with HRP-labeled antibodies specific to mouse IgG (1:1000) or IgA (1:1000) for 1 h at 37 °C. The plates were washed and incubated for 1 h at RT with ABTS (Roche Diagnostics GmbH, Mannheim, Germany). The absorbance was measured at 405 nm on a Jupiter UVM spectrophotometer (ASYS-Hitech GmbH, Eugendorf, Austria). The endpoint titers (EPTs) were expressed as the reciprocal dilution of the last sample dilution of 0.1 OD above the negative control [42,43,44,45].

### 4.9. Statistical Analysis

The results are presented as a mean from each group (*n* = 8) ± standard deviation (SD). Statistical differences between experimental groups were evaluated using ANOVA tests followed by the Tukey post hoc test. A *p*-value of 0.05 was considered significant. Analysis was performed with GraphPad Prism version 8.0.0 for Mac (GraphPad Software, San Diego, CA, USA).

## Data Availability

Not applicable.
